# Image Encryption Method Based on Three-Dimensional Chaotic Systems and V-Shaped Scrambling

**DOI:** 10.3390/e27010084

**Published:** 2025-01-17

**Authors:** Lei Wang, Wenjun Song, Jiali Di, Xuncai Zhang, Chengye Zou

**Affiliations:** 1 Department of Mechanical and Electrical Engineering, Henan Vocational College of Water Conservancy and Environment, Zhengzhou 450002, China; 13838523260@139.com; 2 School of Computer and Communication Engineering, Zhengzhou University of Light Industry, Zhengzhou 450002, China; songwenjun@zzuli.edu.cn; 3 School of Electrical and Information Engineering, Zhengzhou University of Light Industry, Zhengzhou 450002, China; djl3454@163.com; 4 School of Information Science and Engineering, Yanshan University, Qinhuangdao 066004, China

**Keywords:** image encryption, 3D chaotic system, V-shaped scrambling, DNA encoding, DNA manipulation

## Abstract

With the increasing importance of securing images during network transmission, this paper introduces a novel image encryption algorithm that integrates a 3D chaotic system with V-shaped scrambling techniques. The proposed method begins by constructing a unique 3D chaotic system to generate chaotic sequences for encryption. These sequences determine a random starting point for V-shaped scrambling, which facilitates the transformation of image pixels into quaternary numbers. Subsequently, four innovative bit-level scrambling strategies are employed to enhance encryption strength. To further improve randomness, DNA encoding is applied to both the image and chaotic sequences, with chaotic sequences directing crossover and DNA operations. Ciphertext feedback is then utilized to propagate changes across the image, ensuring increased complexity and security. Extensive simulation experiments validate the algorithm’s robust encryption performance for grayscale images, yielding uniformly distributed histograms, near-zero correlation values, and an information entropy value of 7.9975, approaching the ideal threshold. The algorithm also features a large key space, providing robust protection against brute force attacks while effectively resisting statistical, differential, noise, and cropping attacks. These results affirm the algorithm’s reliability and security for image communication and transmission.

## 1. Introduction

Recent advancements in internet technology have revolutionized information exchange and storage in daily life. Increasingly, individuals rely on networks to share resources and achieve interconnectivity, transmitting information in various formats, including text, images, videos, and other multimedia content [1]. Among these, images serve as an intuitive and vivid medium for communication, playing a pivotal role in fields such as politics, economics, and the military [2]. However, the growing prevalence of internet security risks has made images a frequent target for attackers aiming to steal sensitive information [3]. Thus, ensuring the security of image transmission is critically important.

Image encryption algorithms obscure pixel arrangements, transforming them into a disordered state that resembles noise, thereby complicating decryption [4]. Early techniques focused on simple pixel transformations, including Fourier transform [5,6], wavelet transform [7], and Arnold transform [8]. The integration of chaotic systems into encryption algorithms began in 1998 [9], inspired by the Lorenz system’s chaotic unpredictability and sensitivity to initial conditions [10]. This significantly expanded the key space and enhanced algorithm security.

Modern image encryption typically follows a “scrambling-diffusion” framework, leveraging chaotic systems for their randomness, ergodicity, and sensitivity to initial conditions [11,12]. For example, Pak et al. [13] improved the 1D logistic map for faster chaotic behavior, enhancing performance. Similarly, Anandkumar et al. [14] introduced a Henon map-based encryption technique combined with fractal functions. While low-dimensional systems are easy to implement, they often lack sufficient complexity [15]. Hyperchaotic systems, with their richer dynamics, provide greater security and are increasingly favored [16]. For instance, Yang et al. [17] proposed a novel 4D hyperchaotic system using a Liu chaotic system-based flux regulator model. Researchers continue to explore multichaotic systems to improve security and complexity [18,19,20,21].

Scrambling techniques, which rearrange pixel positions, play a key role in encryption [22]. Methods like path scanning, space-filling curves, block scrambling, and fractal scrambling have been extensively studied. For example, Wang et al. [23] combined dynamic row scrambling with zigzag path scanning to enhance efficiency, though challenges remain in handling large-scale images and noise resistance. Advanced approaches, such as Geng et al.’s [24] use of improved Hilbert curve scrambling, offer higher randomness but can suffer from computational complexity. Meanwhile, pixel value replacement methods, such as S-box scrambling [25], face constraints in randomness and effectiveness. Addressing these limitations is essential for advancing encryption methods [26].

The diffusion stage ensures minor image changes propagate throughout the entire image. Yousif et al. [27] combined bit replacement, chaos, and DNA coding to enhance complexity, while Zhang [28] leveraged convolution operations for faster encryption, albeit with reduced security. Other works, such as Zefreh [29], integrated DNA computing with chaotic systems and hash functions to bolster security, though feasibility challenges persist. DNA coding has shown promise, as seen in Almakdi et al.’s [30] circular shifting-based approach, but improvements in processing speed remain a priority.

Building on these advancements, this paper presents an image encryption algorithm that combines a 3D chaotic system with V-shaped scrambling. The algorithm employs SHA-256 hashing to generate a 256-bit value for initializing the chaotic system and producing chaotic sequences. These sequences dictate the random starting point for V-shaped scrambling, followed by quaternary bit-level scrambling. DNA encoding further enhances randomness, with chaotic sequences guiding crossover operations. Ciphertext feedback ensures comprehensive image transformation, completing the encryption process. The main contributions of this paper are as follows.

(1) Improved 3D Chaotic System: A novel system enhances the randomness of chaotic sequences.
(2) Quaternary bit-level scrambling method: Innovative scrambling strategies significantly strengthen encryption.
(3) DNA encoding and chaotic crossover operations: Integration with chaotic sequences increases encryption complexity.

The remainder of this paper is organized as follows: Section 2 discusses the proposed 3D chaotic system. Section 3 details the encryption algorithm, covering key generation, scrambling, DNA encoding, and ciphertext diffusion. Section 4 evaluates the algorithm’s performance. Finally, Section 5 summarizes the contributions and findings.

## 2. Chaotic System

### 2.1. Unified Chaotic System

Can [31] proposes a unified chaotic system by combining Sprott B, Sprott C, van der Schrier–Mass, and Munmuangsaen–Srisuchinwong chaotic flows. Its expression is shown in Equation (1):

(1) $$\left\{\begin{array}{l} \dot{x}=yz\;\;\;\;\;\;\;\;\;\;\\ \dot{y}=\alpha (x-y)\;\;\;\\ \dot{z}=\beta -\gamma xy-(1-\gamma )x^{2} \end{array}\right.$$

where x, y, and z are the input values; $\dot{x}$, $\dot{y}$, and $\dot{z}$ are the output values; and α, β, and γ are control parameters.

### 2.2. Improved 3D Chaotic System

The unified Sprott chaotic system contains three nonlinear terms, two linear terms, and one constant term. To increase the system’s complexity and unpredictability, we incorporate the nonlinear terms sin(*y*) and *yz* into the equation. The system’s expression is given in Equation (2):

(2) $$\left\{\begin{array}{l} \dot{x}=1.3yz+a\sin\left(y\right)\\ \dot{y}=b\left(x-y\right)+0.5yz\\ \dot{z}=c-dxy+(1-d)x^{2} \end{array}\right.$$

where x, y, and z are the state variables of the chaotic system and where *a*, *b*, *c*, and *d* are system parameters that control the chaotic behavior.

**Figure 1:**
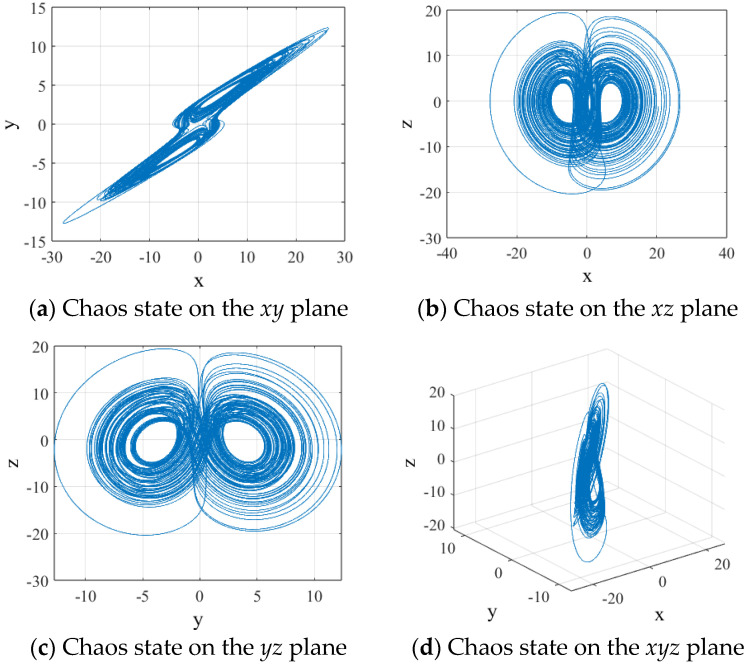
Phase diagrams of the chaotic system.

When the parameters are set as *a* = 1, *b* = 0.95, *c* = 18, and *d* = 0.95 with initial values of *x*_0_ = 0.1, *y*_0_ = 0.1, and *z*_0_ = 0.1, the system exhibits chaotic behavior. Figure 1 displays the phase diagrams of the 3D chaotic system.

### 2.3. Lyapunov Exponent

**Figure 2:**
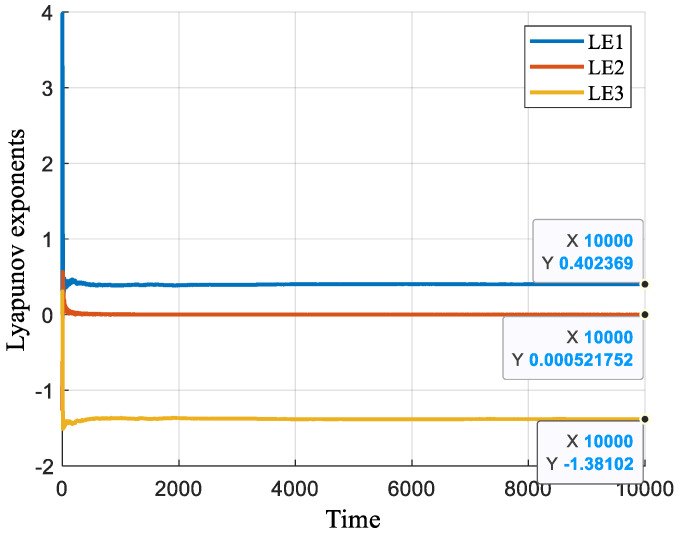
Lyapunov exponent diagram of the chaotic system.

The Lyapunov exponent is crucial for assessing a system’s dynamic characteristics and identifying chaotic behavior. It varies with different parameter values, reflecting the system’s chaotic state. For the proposed 3D chaotic system, the Lyapunov exponents are *LE*_1_ = 0.4024, *LE*_2_ = 0.0005, and *LE*_3_ = −1.3810, as shown in Figure 2. The system is deemed chaotic because it has two positive Lyapunov exponents and the sum of all three exponents is negative.

For a 3D chaotic system, the fractal dimension can be calculated via Equation (3):

(3) $$D_{L}=j+\frac{\sum_{i=1}^{j}{LE}_{i}}{\left|{LE}_{j+1}\right|}=2+\frac{{LE}_{1}+{LE}_{2}}{\left|{LE}_{3}\right|}=2.2917$$

where *j* + 1 represents the system’s dimensionality. A fractal dimension between 2 and 3 indicates chaotic behavior, confirming that the system’s dimension of 2.2917 supports its chaotic nature.

**Figure 3:**
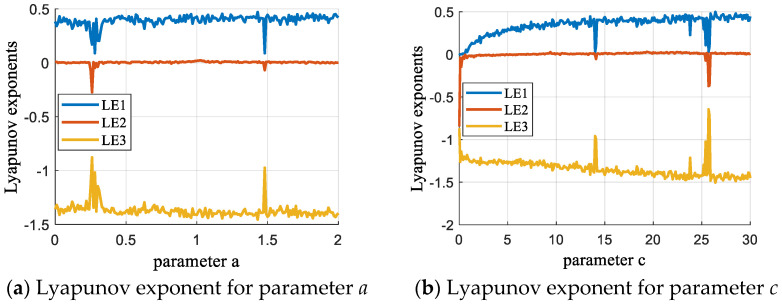
Single-parameter Lyapunov exponent diagrams.

Additionally, the single-parameter Lyapunov exponent plot illustrates the transition from periodic to chaotic behavior in the system. By analyzing this plot, optimal parameter values can be identified to maintain the system in an effective chaotic state. Given the initial state (0.1, 0.1, 0.1), the system parameters are set as shown in Figure 3a,  a ∈ [0,2], b=0.95, c=18, and d=0.95, and in Figure 3b, for a=1, b=0.95, c ∈ [0, 30], and d=0.95.

### 2.4. Bifurcation Diagram Analysis

**Figure 4:**
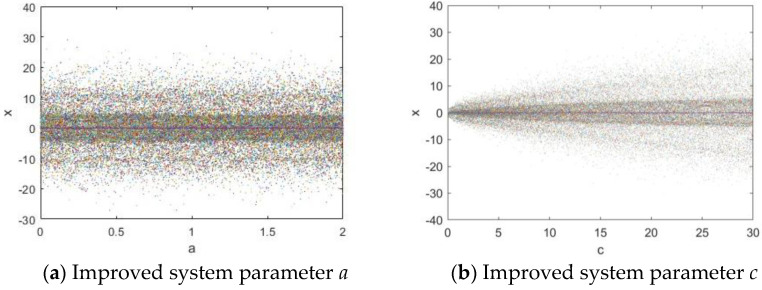
Bifurcation diagrams.

The bifurcation diagram can be used to describe how a system’s behavior changes as the system parameters vary. It not only illustrates the relationship between the steady-state or periodic solutions of a dynamic system and a control parameter but also reveals the system’s transition from order to chaos. Given an initial state of (0.1, 0.1, 0.1), the system parameters are set as follows: in Figure 4a, *a* ∈ [0, 2], *b* = 0.95, *c* =18, and *d* = 0.95; and in Figure 4b, *a* = 1, *b* ∈ [0, 30], *c* = 18, and *d* = 0.95.

### 2.5. Sensitivity Analysis

**Figure 5:**
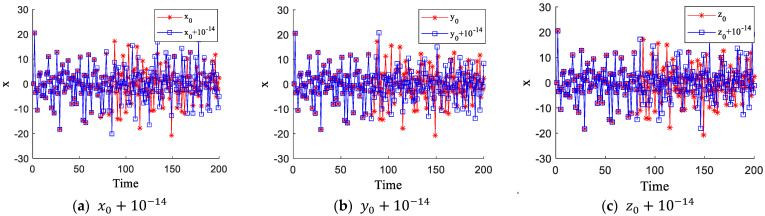
Sensitivity test of the initial values.

A chaotic sequence can be generated by iterating a chaotic system with given initial values. Since chaotic systems are highly sensitive to initial values, even slight changes result in significantly different sequences. In this section, slight variations are applied to the initial values $x_{0}$, $y_{0}$, and $z_{0}$. Three groups of tests were conducted, and the results are shown in Figure 5. The figure demonstrates that even minimal changes to the initial values lead to notable differences in the generated sequences, confirming the proposed 3D chaotic system’s extreme sensitivity to initial values.

### 2.6. NIST Test

The NIST randomness test is an industry-standard method for evaluating the randomness of generated sequences. In this experiment, the NIST test was applied to sequences from the 3D chaotic system. The parameters were set as per Table 1, and the decimal sequences were converted to binary sequences for testing.

The NIST test, comprising 15 subtests, deems a sequence random if the *p* value exceeds 0.01. Each sequence was tested with 30 samples of 1,000,000 binary digits each. Table 2 shows that all subtests achieved *p* values above 0.01, validating the randomness of the chaotic sequence and confirming the security of the encryption.

## 3. Image Encryption Scheme

**Figure 6:**
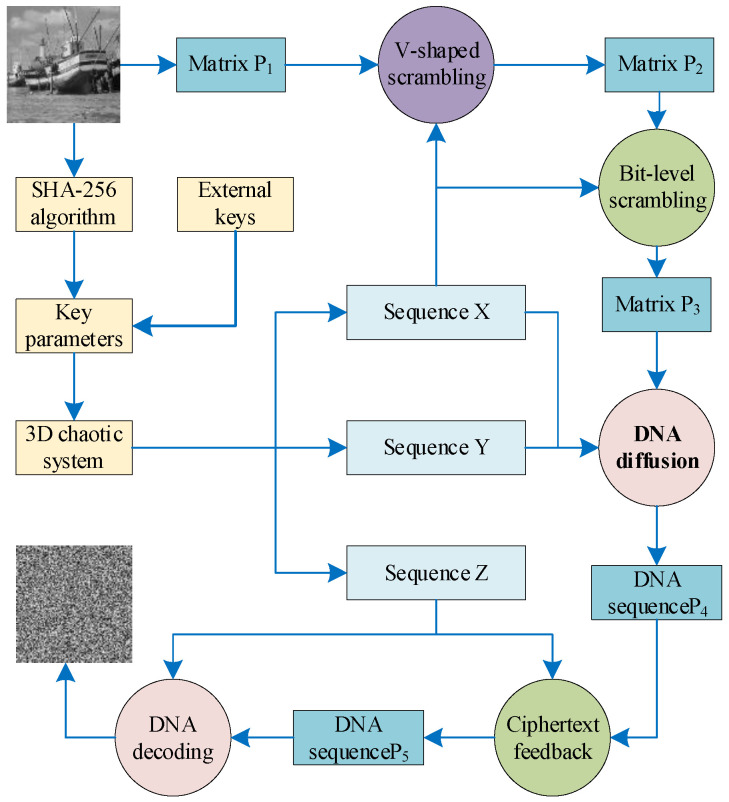
Flowchart of the image encryption method.

The encryption process starts with V-shaped pixel scrambling, followed by bit-level scrambling using a quaternary-based approach. Next, DNA encoding and additional scrambling are performed based on the proposed DNA method. Finally, ciphertext diffusion ensures secure and efficient encryption. The flowchart of the process is shown in Figure 6.

### 3.1. Initialization of the Chaotic System

The input image is processed using the SHA-256 algorithm, generating a 256-bit binary hash value *K*, which serves as the external key. This key is divided into 32 groups, each containing an 8-bit binary number, *K* = {*K*_1_, *K*_2_, …, *K*_32_}. The key is further processed using Equation (4):

(4) $$\left\{\begin{array}{c} \begin{array}{c} \alpha =\frac{mod(\left(K_{1}+K_{2}+K_{3}+K_{4}\right),256)⨁mod(\left(K_{5}+K_{6}+K_{7}+K_{8}\right),256)}{256}\\ \beta =\frac{mod(\left(K_{9}+K_{10}+K_{11}+K_{12}\right)⨁{mod((K}_{13}+K_{14}+K_{15}+K_{16}),256)}{256} \end{array}\\ \gamma =\frac{mod(\left(K_{17}+K_{18}+K_{19}+K_{20}\right)⨁mod(\left(K_{21}+K_{22}+K_{23}+K_{24}\right),256)}{256}\\ \sigma =\frac{mod(\left(K_{25}+K_{26}+K_{27}+K_{28}\right)⨁mod(\left(K_{29}+K_{30}+K_{31}+K_{32}\right),256)}{256} \end{array}\right.$$

where ⊕ represents the XOR operation. The initial values for the 3D chaotic system are subsequently determined via Equation (5):

(5) $$\left\{\begin{array}{c} x_{0}=mod\left(\left(\alpha +\beta \right),1\right)+x_{0}^{\prime }\\ y_{0}=mod\left(\left(\beta +\gamma \right),1\right)+y_{0}^{\prime }\\ z_{0}=mod\left(\left(\gamma +\sigma \right),1\right)+z_{0}^{\prime } \end{array}\right.$$

where $x_{0}^{\prime }$, $y_{0}^{\prime }$, and $z_{0}^{\prime }$ are the external keys.

The key generation process, which combines the image hash value with an external key, has both strengths and limitations. On the one hand, this approach creates a dynamic key that enhances security, making it highly resistant to reverse-engineering attacks. On the other hand, the need to transmit the image hash value and external key introduces a potential vulnerability. Although the hash value is significantly smaller than the entire image, its secure transmission remains a challenge and bears similarities to the key distribution issues encountered in traditional one-time pad (OTP) schemes. Despite being less efficient in key management compared to methods solely relying on external keys, this approach strikes a practical balance between ensuring robust cryptographic security and maintaining usability.

### 3.2. V-Shaped Scrambling

Scrambling rearranges image pixels to obscure their structure, making information appear random and inaccessible. V-shaped scrambling introduces a novel approach, enhancing randomness and security.

For an image matrix *P* of size *M* × *N*, each time, *N* pixels are traversed and denoted as ${T=(t}_{1},t_{2},\ldots t_{N})$, which are then swapped with the pixels in the last row of the matrix. The detailed process is as follows:

(1) Select a random starting pixel: A starting pixel *t*_1_ = *P_k_*, where 1 ≤ k ≤ M, is randomly chosen from the first column of the matrix.

(2) Pixel traversal (the explanation below is for the case where *M* ≤ *N*; for *M* > *N*, simply adjust the traversal order):

When *M* = *N*: Starting from the initial position (*k*, 1), move diagonally downward until reaching the last row, then move diagonally upward, selecting pixels along the way until reaching the last column. The selected pixels are as follows:

(6) $$t_{j}=\left\{\begin{array}{c} P_{k+j-1,j},\;\;\;\;1\leq j\leq M-k+1\\ P_{2\times M-j-k+1,j},\;\;\;M-k+1<j\leq N \end{array}\right.$$

When *M* < *N*: Similarly to the *M = N* case, move diagonally downward until reaching the last row, then move diagonally upward. If the number of traversed elements is less than *N*, the process is repeated from the current pixel as the starting point until *N* pixels are traversed.

(3) Pixel swapping: Each traversed pixel $t_{j}$ is swapped with the corresponding pixel in the last row of the matrix $P_{M,j}$.

(4) Matrix update: After completing one traversal and swapping, save the current last row, reduce the number of rows *M* by 1 (i.e., set *M* = *M* − 1), and repeat the above steps until all the pixels are traversed and swapped. A total of *N* − 1 traversals are needed.

Through this process, the pixels of the image are rearranged and swapped along the V-shaped path, achieving scrambling and enhancing the encryption strength and confidentiality of the image.

**Figure 7:**
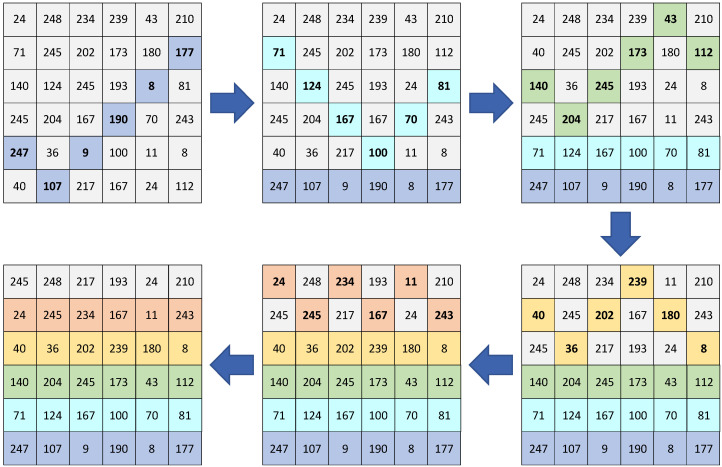
Schematic diagram of V-shaped scrambling.

For a 6 × 6 matrix *P*, five traversals are required. Starting positions are chosen randomly, and pixels are swapped row by row, gradually reducing the matrix size. The full process is illustrated in Figure 7.

### 3.3. Bit-Level Scrambling

After pixel-level scrambling, the image data remain unchanged, making it vulnerable to brute force attacks. To enhance security, this paper introduces a quaternary-based bit-level scrambling method that fully scrambles the pixel bit distribution.

**Figure 8:**
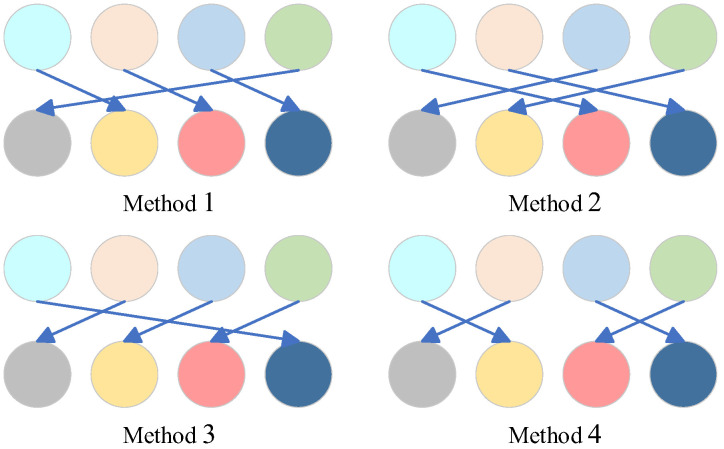
Quaternary number scrambling method.

Pixels are converted into quaternary values via four-digit sequences. Figure 8 shows four possible conversion methods, where each sequence pair represents the plain image’s quaternary sequence and the corresponding chaotic sequence.

**Figure 9:**
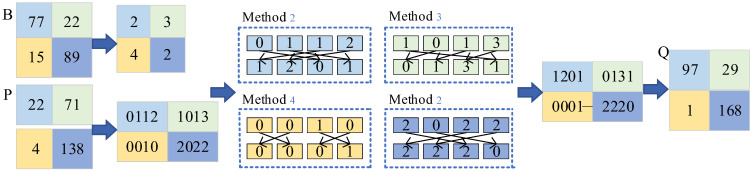
Schematic diagram of bit-level scrambling.

Figure 9 illustrates the bit-level scrambling process. The matrix *B* selects the conversion method, *P* represents the plain image, and *C* is the chaotic sequence matrix. *P* is converted into quaternary forms, and the matrix *B* determines the conversion mode. For example, the upper-left corner of *P* is converted via Method 2 (Figure 9), which transforms “0112” into “1201” and “0133” into “3301”.

### 3.4. DNA Diffusion

The diffusion process is critical for ensuring that changes to a single plaintext pixel or key affect every pixel in the image, thus enhancing key sensitivity and providing defense against differential attacks. While pixel-level scrambling conceals visual information, it does not modify the statistical characteristics of the image, which can pose security risks. DNA diffusion aims to leverage the unique properties of DNA molecules to increase the security and efficiency of encryption. The complexity and unpredictability of DNA sequences provide stronger resistance against attacks for encryption algorithms.

#### 3.4.1. DNA Encoding

The process typically involves converting image pixels into binary representations, with the binary values ‘00’, ‘01’, ‘10’, and ‘11’ corresponding to the four bases in DNA. Following this, the binary sequence is transformed into a DNA sequence, which is then scrambled, substituted, or obfuscated to create a key that strengthens the encryption’s security. The coding rules are summarized in Table 3.

#### 3.4.2. DNA Diffusion Operation

**Figure 10:**
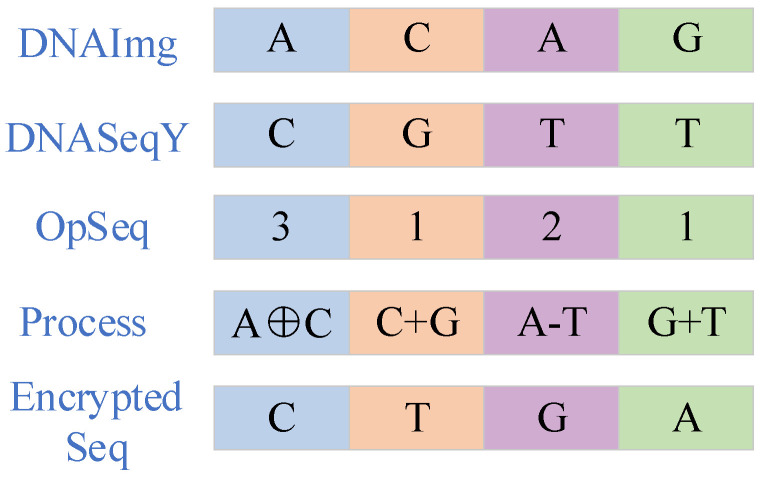
Schematic diagram of DNA diffusion.

The operational rules for DNA diffusion are outlined in Table 4, Table 5 and Table 6. In image encryption, by converting image pixels into DNA sequences and utilizing the base pairing rules of DNA, the randomness and security of the encryption effectively increase. In the DNA encoding method, the original image and chaotic sequences are encoded via randomly selected DNA rules. The chaotic sequence plays a pivotal role in controlling this encoding process: OpSeq (1–5) specifies the type of DNA operation to be executed (addition, subtraction, XOR, or multiplication). The diffusion diagram is shown in Figure 10.

This structured approach not only enhances encryption effectiveness but also aligns with contemporary cryptographic applications, underscoring the significance of DNA-based techniques in modern security frameworks.

### 3.5. Ciphertext Feedback

In ciphertext feedback mode, the previous ciphertext block is used as input for encrypting the next plaintext block, creating a stream encryption mode suitable for real-time communication or network transmission. This feedback mechanism introduces randomness, increasing cryptosystem security by preventing common symmetric encryption attacks.

Ciphertext feedback is performed via Equation (7):

(7) $$q_{i}=\left\{\begin{array}{c} p_{i}⊕E_{i}\;\;\;\;\;\;\;\;\;\;\;\;\;\;\;\;\;\;\;\;\;\;\;\;\;\;\;\;i=1\\ \left(p_{i}⊕q_{i-1}\right)⊕E_{i}\;\;\;\;\;\;\;\;\;\;\;\;i\geq 1 \end{array}\right.$$

where *p_i_* is the plain image DNA sequence, *E_i_* is the chaotic DNA sequence, and *q_i_* is the resulting ciphertext sequence; *i* =1, 2…4 × *M* × *N*.

### 3.6. Encryption Process

The encryption process is applied to a grayscale image *P* of size *M* × *N* and follows these steps below.

Step 1: Convert the grayscale image *P* into a matrix *P*_1_ of size *M* × *N* and then apply the SHA-256 algorithm to generate the hash value *K*. Use Equations (4) and (5) to compute the initial values *x*_0_, *y*_0_, and *z*_0_.

Step 2: Substitute the initial values *x*_0_, *y*_0_, and *z*_0_ into the 3D chaotic system, iterate 2 × *M* × *N* + *N*0 times, discard the first *N*_0_ iterations to eliminate the transient effects in the chaotic sequence, and obtain chaotic sequences *X*, *Y,* and *Z* with a length of 2 × *M* × *N*.

Step 3: Split the chaotic sequence *X* into two sequences *A* and *B* of length *M* × *N*; split the chaotic sequence *Y* into two sequences C and D of length *M* × *N*; split the chaotic sequence *Z* into two sequences *E* and *F* of length *M* × *N*; and then concatenate the chaotic sequences *X* and *Y* to form a new chaotic sequence *G* with a length of 4 × *M* × *N*.

Step 4: Sort sequence *A* in ascending order to obtain the index vector *A*_index_ and use this index to perform simple scrambling on the image matrix *P*_1_. Take the first *M* − 2 values of sequence *B*, apply formula (8) to process them, and obtain a new sequence *B*′, which is used to randomly select the starting position for V-shaped scanning. V-shaped scrambling is performed on *P*_1_ to obtain the scrambled image matrix *P*_2_.

(8) $$B\prime \left(i\right)=mod\left(\left\lfloor B\left(i\right)\times 10^{10}\right\rfloor ,\;M-i\right)+1,\;i=1,2,\ldots ,M-2;$$

Step 5: Process the chaotic sequence *B* via Equation (9). The matrix *P*_2_ is converted into the quaternary form. The conversion mode for each pixel is randomly selected on the basis of the processed result, and the conversion is performed via the proposed bit-level scrambling method. The image matrix *P*_2_ is scrambled to obtain the scrambled image matrix *P*_3_.

(9) $$Trans\left(i\right)=mod\left(\left\lfloor B\left(i\right)\times 10^{10}\right\rfloor ,\;4\right)+1,\;\;i=1,2\ldots ,M\times N;$$

Step 6: Apply formula (10) to process sequences *C* and *D*, obtaining sequences *R*′ and *D*′, respectively. The DNA encoding rule *R*′ is selected to encode *P*_3_ and the chaotic sequence *D*′ into the DNA sequences *DNAImg* and *DNASeq*, respectively.

(10) $$\left\{\begin{array}{c} R\prime \left(i\right)=mod\left(\left\lfloor C\left(i\right)\times 10^{10}\right\rfloor ,\;8\right)+1\\ D^{\prime }\left(i\right)=mod\left(\left\lfloor D\left(i\right)\times 10^{10}\right\rfloor ,\;256\right) \end{array}\right.,\;i=1,2,\ldots ,M\times N$$

Step 7: Process sequence *G* according to Equation (11) to obtain sequence *OpSeq*; perform diffusion processing on *DNAImg* and *DNASeq*, according to the DNA diffusion algorithm to obtain sequence *P*_4_.

(11) $$OpSeq\left(i\right)=mod\left(Z,5\right)+1,\;i=1,2,\ldots ,4\times M\times N;$$

Step 8: Apply formula (12) to process sequence *E* and obtain sequences *F*′. Perform ciphertext feedback *P_4_* via Equation (9) to generate sequence *P_5_*.

$$D^{\prime }\left(i\right)=mod\left(\left\lfloor D\left(i\right)\times 10^{10}\right\rfloor ,256\right)$$

Step 9: Process sequence *G* according to Equation (14) to dynamically select the DNA encoding rules, apply the DNA decoding process to sequence *P*_5_, and then reconstruct it into the image matrix *P*_6_ of size *M* × *N.*

(12) $$r_{3}\left(i\right)=mod\left(G\left(i\right),8\right)+1,\;i=1,2,3\ldots ,M\times N;$$

The decryption process is the inverse of the encryption process, applying the reverse operations in sequence.

## 4. Security Analysis

To evaluate the security and reliability of the proposed algorithm, simulation experiments were conducted using the USC-SIPI image database and standard test images implemented in MATLAB R2018a on a Windows 10 platform. The experiment sets the external keys $x_{0}^{\prime }=0.01$, $y_{0}^{\prime }=0.01$, and $z_{0\;}^{\prime }$= 0.01.

**Figure 11:**
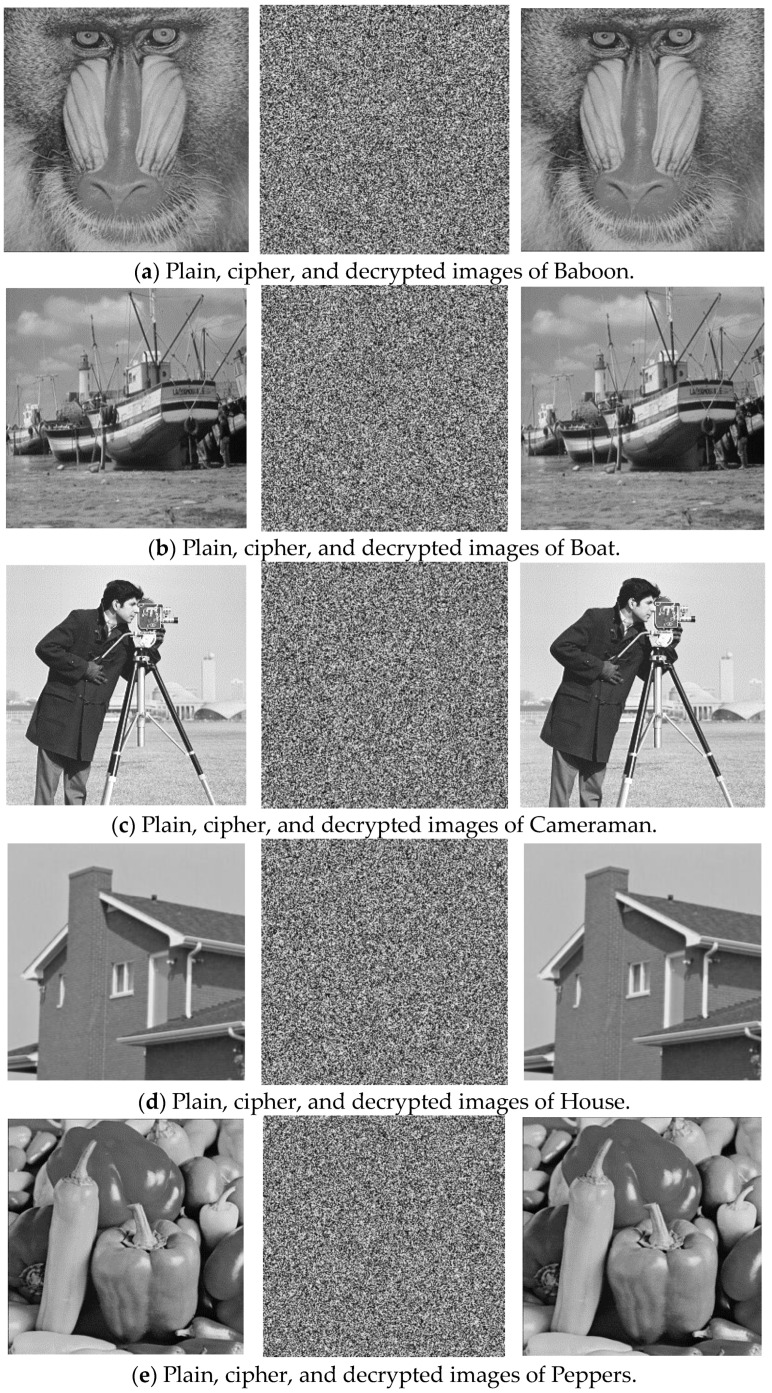
Encryption and decryption results.

Five images, each with a resolution of 256 × 256, were encrypted. The results are shown in Figure 11. The encrypted images appear noisy, whereas the decrypted images match the original images exactly, confirming the effectiveness of the encryption algorithm.

### 4.1. Key Space Analysis

The key space refers to the total number of possible keys in the encryption algorithm, with larger key spaces offering greater resistance to brute force attacks. The key space of the proposed algorithm is 10^90^, far exceeding the standard threshold of 2^100^, providing strong defense against exhaustive search methods.

### 4.2. Key Sensitivity Analysis

Key sensitivity measures how small variations in the encryption key influence the initial values and, in turn, the encryption results. High sensitivity ensures that even the slightest key changes produce vastly different encrypted outputs, making decryption without the correct key result in images completely unlike the original. This property is crucial for evaluating the robustness of the encryption algorithm.

In this analysis, only one key parameter is changed while the others remain constant. Table 7 lists the key variations used for this analysis.

(1) Key sensitivity analysis during encryption

**Figure 12:**
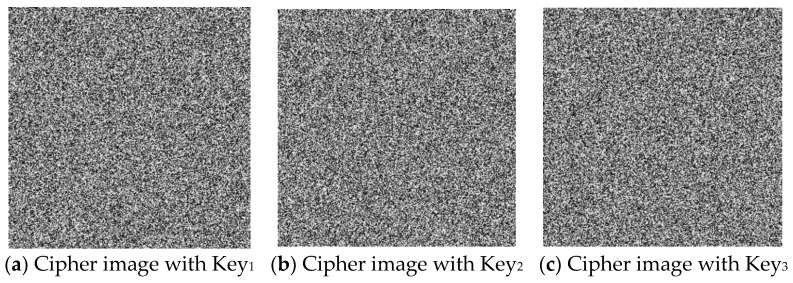
Sensitivity analysis for the encryption process.

The “Boat” image was encrypted via the key sets outlined in Table 7, with the resulting encrypted images displayed in Figure 12. Although the visual differences between the encrypted images may appear subtle, metrics such as the number of pixels change rate (NPCR) and unified average changing intensity (UACI) provide a more precise evaluation of these variations.

The NPCR quantifies the percentage of pixels that differ between two images, whereas the UACI measures the average intensity of pixel changes relative to the maximum possible difference. Together, the NPCR and UACI provide a comprehensive assessment of pixel changes between encrypted images produced with different keys. Under ideal conditions, the NPCR should be 99.6094% and the UACI should be 33.4635%, which is calculated as follows:

(13) $$NPCR=\frac{\sum_{i,j}D\left(i,j\right)}{M\times N}\times 100\%$$

(14) $$UACI=\frac{1}{M\times N}\frac{\sum \left(C_{1}\left(i,j\right)-C_{2}\left(i,j\right)\right)}{255}\times 100\%$$

where *M* and *N* indicate image width and height, respectively, and D(*i*, *j*) is defined as:

(15) $$D\left(i,j\right)=f\left(x\right)=\left\{\begin{array}{l} 1,\;\;\;\;\;\;\;\;C_{1}\left(i,j\right)\neq C_{2}\left(i,j\right)\\ 0,\;\;\;\;\;\;\;\;otherwise \end{array}\right.$$

The results in Table 8 reveal that the NPCR and UACI values are near the ideal targets, highlighting the algorithm’s high sensitivity to key changes. A minor variation in the encryption key leads to a notably different output, showcasing its strong security.

(2) Key sensitivity analysis during decryption

**Figure 13:**
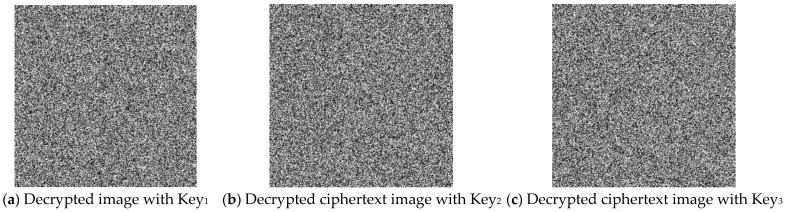
Key sensitivity analysis for the decryption process.

The “Boat” cipher image was decrypted via the key sets in Table 7. The results, shown in Figure 13, demonstrate that even slight key variations prevent correct decryption, thereby enhancing the algorithm’s security. The key sensitivity analysis confirms that the proposed method is highly sensitive in both the encryption and decryption phases, making it secure against key-related attacks.

### 4.3. Statistical Analysis

#### 4.3.1. Histogram Analysis

**Figure 14:**
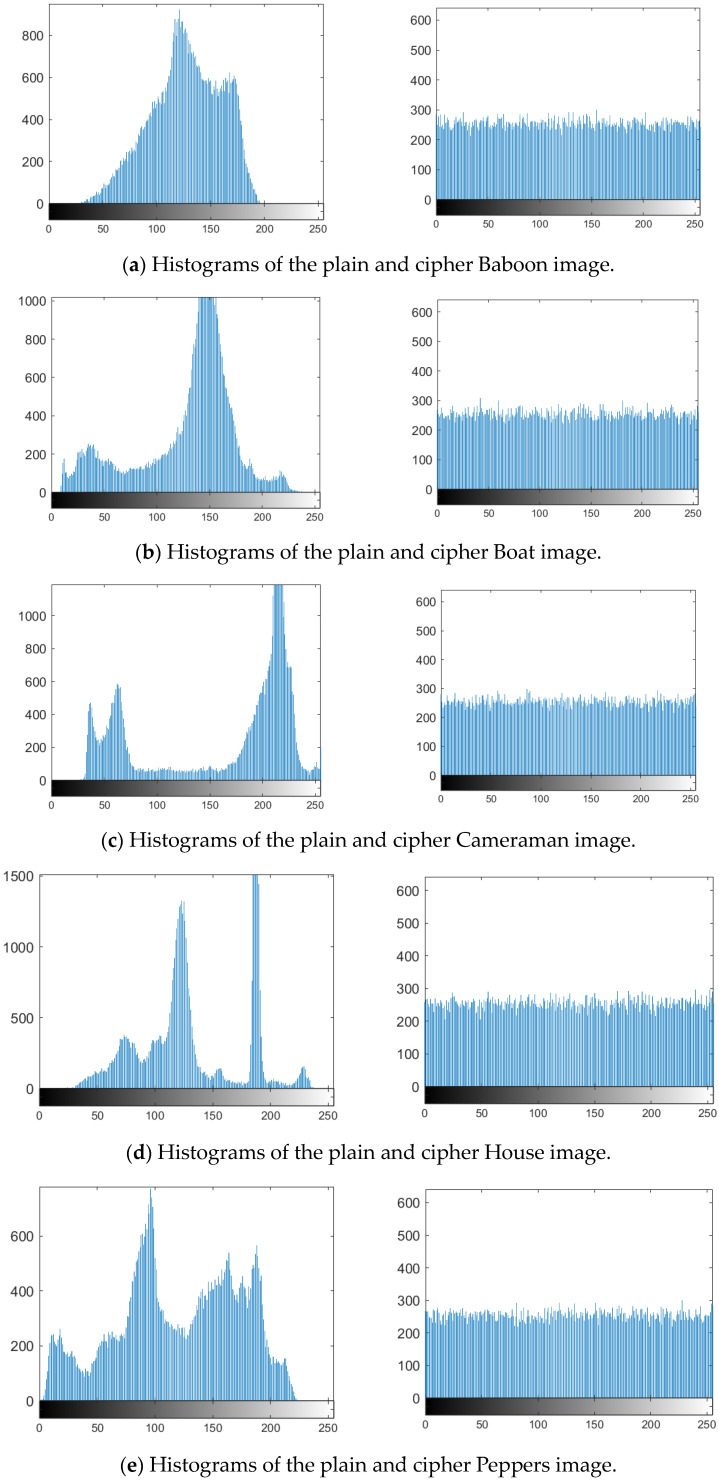
Image histogram analysis results.

The histogram of an image displays the distribution of pixel values and is a key measure of an encryption algorithm’s ability to resist statistical attacks. Plain images often exhibit patterns that can be exploited by attackers, making it essential for the ciphertext to effectively obscure these patterns. A strong encryption algorithm should produce a ciphertext with a nearly uniform histogram. As illustrated in Figure 14, the histogram of the encrypted image is nearly uniform, in contrast to the uneven distribution of the original image, demonstrating that the algorithm successfully conceals the underlying data.

#### 4.3.2. Correlation Analysis

**Figure 15:**
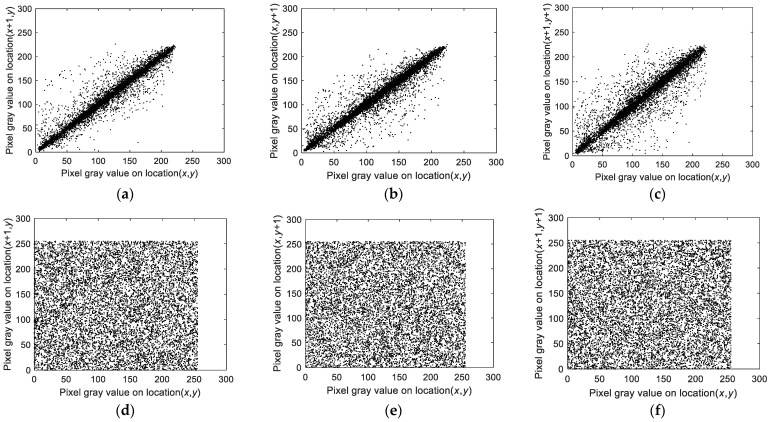
Correlations of adjacent pixels of the image before and after encryption. (**a**) Horizontal of the plain image; (**b**) vertical of the plain image; (**c**) diagonal of the plain image; (**d**) horizontal of the encrypted image; (**e**) vertical direction of the encrypted image; (**f**) diagonal of the encrypted image.

For enhanced security, an encryption algorithm should significantly lower the correlation between adjacent pixels. High pixel correlation in ciphertext may reveal clues that compromise the encryption. The correlation coefficient, which indicates the strength of the relationship between adjacent pixels, should be close to 0 for more effective encryption. The correlation coefficient, which is calculated via Equation (16), is detailed in Table 9 and visualized in Figure 15 for the Peppers image.

(16) $$\left\{\begin{array}{l} r_{x,y}=\frac{cov\left(x,y\right)}{\sqrt{D\left(x\right)D\left(y\right)}}\\ cov\left(x,y\right)=E\left(\left[x-E\left(x\right)\right]\left[y-E\left(y\right)\right]\right)\\ E\left(x\right)=\frac{1}{N}\sum_{i=1}^{N}x_{i}\\ D\left(x\right)=\frac{1}{N}\sum_{i=1}^{N}{\left[x_{i}-E\left(x\right)\right]}^{2} \end{array}\right.$$

Here, *x* and *y* represent pairs of adjacent pixels, *E* (*x*) and *E* (*y*) are the expected values of the pixel intensities, *D* (*x*) and *D* (*y*) are the variances, and *N* is the number of pixel pairs in the sample.

Table 9 shows that the correlation coefficients for the cipher images are near 0. Figure 15 further illustrates a marked reduction in correlation in the scatter plots. These results confirm that the encryption algorithm effectively reduces pixel correlation, thus enhancing image security.

### 4.4. Resistance to Differential Attacks

Differential attacks aim to exploit small modifications in the original image to analyze their impact on the corresponding encrypted outputs. By comparing two encrypted images—one from the original version and the other from a slightly altered version—attackers may attempt to detect patterns that could compromise the encryption. To prevent this, the encryption algorithm must produce vastly different encrypted outputs even with minor changes in the original image.

The algorithm’s resilience to differential attacks is assessed by calculating the NPCR and UACI between two encrypted images that differ by only one pixel. As shown in Table 10, the results obtained via Equations (13) and (14) confirm that the proposed encryption algorithm is highly sensitive to small changes in the plaintext, providing robust protection against differential attacks.

### 4.5. Noise Attack

A noise attack involves interference with the encrypted image during transmission, potentially leading to disturbances or information loss that complicate decryption. Evaluating the encryption algorithm’s resistance to noise attacks is crucial for assessing its robustness [32].

**Figure 16:**
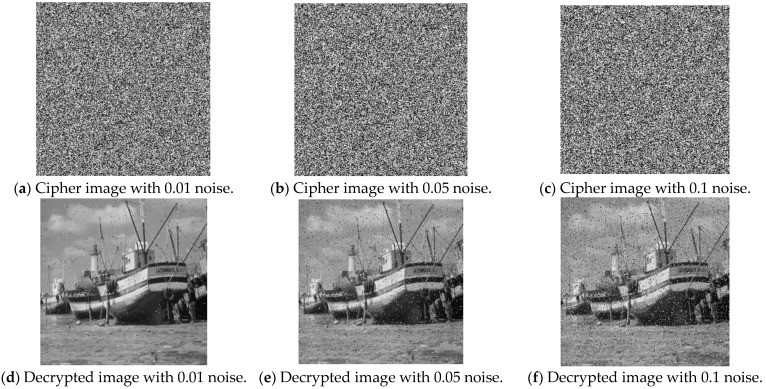
Results of salt and pepper noise attack.

To evaluate noise resistance, varying levels of salt-and-pepper and Gaussian noise are added to the encrypted image, followed by visual comparison of the decrypted image with the original image to assess restoration effectiveness. Figure 16a–c display the encrypted “Boat” images with salt-and-pepper noise at densities of 0.01, 0.05, and 0.1, respectively, whereas Figure 16d–f show the corresponding decrypted images. Despite noise interference, the decrypted images retain enough detail to recognize the original content.

A quantitative analysis of noise resistance is performed via the peak signal-to-noise ratio (PSNR) and mean square error (MSE) metrics. The PSNR evaluates the similarity between the original and decrypted images, with higher PSNR values indicating stronger resilience against noise. The PSNR is calculated as follows:

(17) $$\left\{\begin{array}{l} MSE=\frac{1}{M\times N}\sum_{i=1}^{M}\sum_{j=1}^{N}{\left[P\left(i,j\right)-D\left(i,j\right)\right]}^{2}\\ PSNR=20\log_{10}\left(\frac{255}{\sqrt{MSE}}\right)\;\;\;\;\;\;\;\;\;\;\;\;\;\;\;\;\;\;\;\;\;\;\;\;\;\;\; \end{array}\right.$$

where *M* and *N* represent the dimensions of the image, and *P*(*i*, *j*) and *D*(*i*, *j*) denote the pixel values of the original and decrypted images, respectively.

The PSNR values under different noise densities are presented in Table 11, highlighting the algorithm’s strong resistance to salt-and-pepper noise interference.

### 4.6. Cropping Attack

**Figure 17:**
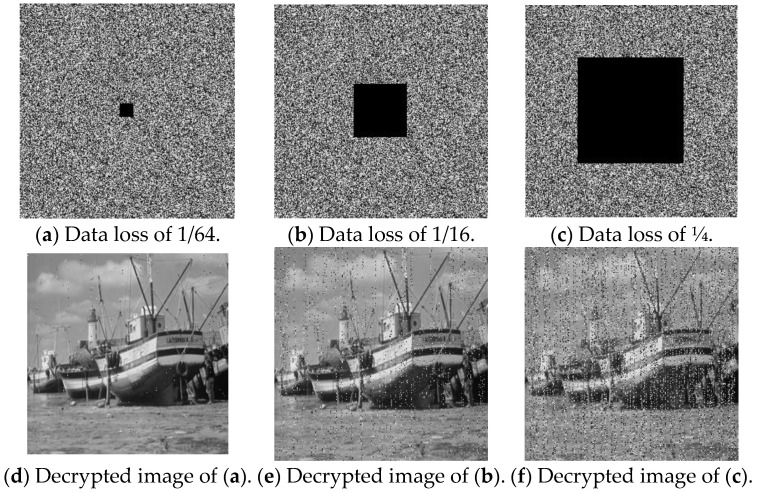
Results of cropping attacks.

During image transmission, data loss may occur, leading to incomplete image information. A robust image encryption algorithm should be capable of restoring the original image even after cropping attacks of varying severity [33]. As depicted in Figure 17a–c, the encrypted image undergoes cropping by 1/64, 1/16, and 1/4, respectively, and these cropped encrypted images are subsequently decrypted. The decrypted results are shown in Figure 17d–f, illustrating that the key features of the original image remain recoverable despite the cropping attack.

The PSNR values comparing the original image with the decrypted image after the cropping attack are presented in Table 12. The analysis indicates that the encryption algorithm demonstrates strong resilience against cropping attacks.

### 4.7. Chi-Square Test

The chi-square test is used to assess the distribution of pixel intensity values in both the original image and the encrypted image. Essentially, it calculates the frequency of pixel values in a grayscale image to determine whether the pixel distribution is uniform [34]. A larger chi-square value indicates a more uneven pixel distribution. According to chi-square test theory, for images with gray levels ranging from 0 to 255, the critical value for the chi-square test is 293.2478. The chi-square test is calculated via the following formula:

(18) $$\chi^{2}=\sum_{j=0}^{255}\frac{{\left(P_{i}-P_{e}\right)}^{2}}{P_{e}}$$

(19) $$P_{e}=\frac{M\times N}{I_{max}}$$

where $P_{i}$ represents the actual frequency of occurrence of pixel *i*, $P_{e}$ is the expected frequency, *M* and *N* are the image dimensions, and $I_{max}$ is the total number of pixel intensities in the grayscale image.

Table 13 presents the chi-square values for both the plain and cipher images. The chi-square values of the cipher images are clearly all below the critical value, whereas the chi-square value for the plain image is large, indicating that the encryption algorithm effectively encrypts the image, ensuring that the pixel values in the cipher image are evenly distributed.

### 4.8. Information Entropy and Local Information Entropy

Information entropy measures the level of uncertainty and randomness in an image, which reflects the effectiveness of the encryption. It calculates the average information content after accounting for redundancy and indicates the encryption system’s confusion level. The entropy is given as

(20) $$H=-\sum_{x=1}^{N}p\left(x\right)\log_{2}\left(p\left(x\right)\right)$$

where *p* (*x*) is the probability of pixel value *x* and *N* is the number of gray levels in the image (256). The theoretical maximum entropy value for a perfectly encrypted image is 8. The values close to 8, as shown in Table 14, suggest that the information is well distributed, indicating effective encryption.

Local information entropy analysis is performed by dividing the image into regions, computing the entropy for each region, and then integrating the results into an overall information distribution map [35]. Table 15 shows that the entropy values are near 8, confirming the encryption algorithm’s high security.

### 4.9. Known-Plaintext and Chosen-Plaintext Attacks

Data loss can occur during image transmission, but a strong encryption algorithm can recover the original image even after cropping attacks [36]. Each key in this algorithm is unique to the input image, making it resistant to known-plaintext and chosen-plaintext attacks [37].

In a chosen-plaintext attack, encrypting all-white or all-black images reveals minimal transformation, as the pixel values are limited to 0 or 255. Figure 16 and Figure 17 show the encrypted images, their histograms, and the correlation of adjacent pixels, demonstrating effective concealment of visual information.

**Figure 18:**
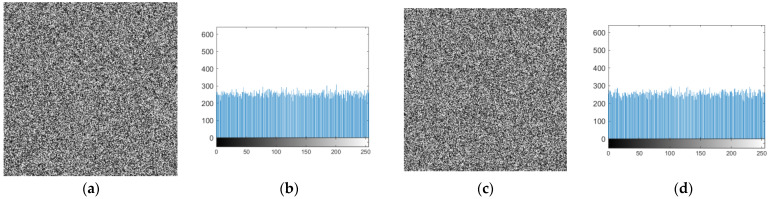
Encryption results and histogram analysis of all-black and all-white images. (**a**) Encrypted all-black image; (**b**) histogram of encrypted all-black image; (**c**) encrypted all-white image; (**d**) histogram of encrypted all-white image.

**Figure 19:**
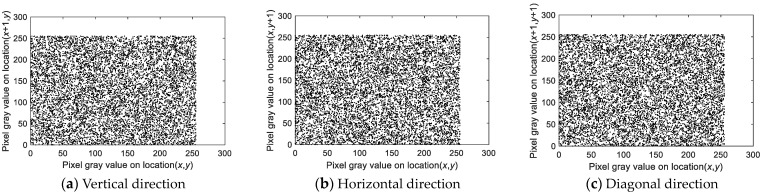
Correlation analysis of the encrypted all-black image.

As observed in Figure 18 and Figure 19, the histogram distribution of the encrypted images is highly uniform, and the correlation distribution forms a scatter plot, demonstrating that even for all-black and all-white images, the encryption algorithm effectively conceals visual information, converting it into a uniform, undecipherable format. The quantitative analysis results in Table 16 and Table 17 further confirm that the encryption algorithm reduces adjacent pixel correlations to nearly zero and achieves information entropy close to the ideal value, ensuring robust defense against differential attacks.

### 4.10. Comparative Analysis

The proposed encryption algorithm was compared with others via a 256 × 256 Pepper image. Table 18 shows that it outperforms other algorithms in terms of correlation, information entropy, NPCR, and UACI, demonstrating superior robustness and security. The algorithm, which uses a 3D chaotic system and V-shaped scrambling, offers enhanced security and is suitable for various image security applications.

### 4.11. Time Complexity Analysis

Time complexity is a key metric for evaluating the efficiency of algorithms. For a grayscale image of size *M* × *N*, the time complexity of V-shaped scrambling is O (*M* × *N*), while both bit-level scrambling and the DNA diffusion step also have a complexity of O (*M* × *N*). Consequently, the overall encryption time complexity is O (6 *M* × *N*). Typically, bit-plane scrambling operates in an 8-bit binary mode with a complexity of O (8 *M* × *N*), but in this paper, it is executed on a quaternary sequence with a complexity of O (4 *M* × *N*), which effectively enhances the encryption efficiency.

To provide a more intuitive demonstration of the encryption performance, we conducted timing experiments on a hardware platform equipped with MATLAB R2018a. We compared the running time of our algorithm against those of other established algorithms, and the results are summarized in Table 19. The experimental results indicate that our algorithm has significant advantages in terms of encryption efficiency, allowing rapid image encryption processing.

These results demonstrate the superior performance of our proposed method in both encryption and decryption, reinforcing its practical applicability in real-time scenarios.

## 5. Conclusions

In this paper, we propose an image encryption algorithm that processes the plaintext image using the SHA-256 algorithm to generate a 256-bit hash value. This hash value serves as the initial condition for the chaotic system, which is subsequently input into a 3D chaotic model to generate a chaotic sequence used in the encryption process. During pixel scrambling, the starting position for the V-shaped scrambling is determined randomly by the chaotic sequence, enabling efficient pixel rearrangement. The image and chaotic sequence are then encoded into DNA sequences, where the chaotic sequence controls both the crossover and DNA operations between the image’s DNA sequence and the chaotic DNA sequence. Finally, ciphertext feedback is applied to propagate changes across the entire image, increasing the complexity and security of the encryption.

Testing on various images demonstrated that the encrypted outputs exhibited uniformly distributed histograms, near-zero correlation coefficients, and an entropy value of 7.9975, indicating strong resistance to brute force, statistical, differential, noise, and cropping attacks. The scheme also benefits from a large key space, further enhancing its robustness against cryptanalysis. 

## Figures and Tables

**Table 1:** NIST random test parameters.

Parameters	Value
Within-block frequency test	128
Nonoverlapping module matching test	9
Duplicate module matching test	9
Approximate entropy test	10
Sequence test	16
Linear complexity test	500

**Table 2:** Results of the NIST random test.

NIST Test Items	Results			
	*x*	*y*	*z*	Pass/Fail
Frequency	0.350485	0.407091	0.468595	Pass
Block Frequency	0.804337	0.066882	0.862344	Pass
Runs	0.911413	0.299251	0.739918	Pass
Longest Run	0.213309	0.178278	0.299251	Pass
Rank	0.739918	0.213309	0.054199	Pass
FFT	0.976060	0.407091	0.976060	Pass
Nonoverlapping Template	0.100508	0.468595	0.213309	Pass
Overlapping Template	0.534146	0.602458	0.082177	Pass
Universal	0.671779	0.911413	0.054199	Pass
Linear Complexity	0.253551	0.407091	0.804337	Pass
Serial	0.671779	0.949602	0.862344	Pass
Approximate Entropy	0.148094	0.739918	0.949602	Pass
Cumulative Sums	0.739918	0.253551	0.602458	Pass
Random Excursions	0.025193	0.162606	0.392456	Pass
Random Excursions Variant	0.090936	0.275709	0.788728	Pass

**Table 3:** DNA encoding rules.

Rules	1	2	3	4	5	6	7	8
00	A	A	T	T	C	C	G	G
01	G	C	G	C	T	A	T	A
10	C	G	C	G	A	T	A	T
11	T	T	A	A	G	G	C	C

**Table 4:** DNA addition.

+	A	G	C	T
A	A	G	C	T
G	G	C	T	A
C	C	T	A	G
T	T	A	G	C

**Table 5:** DNA subtraction.

−	A	G	C	T
A	A	T	C	G
G	G	A	T	C
C	C	G	A	T
T	T	C	G	A

**Table 6:** DNA XOR operation.

⊕	A	G	C	T
A	A	G	C	T
G	G	A	T	C
C	C	T	A	G
T	T	C	G	A

**Table 7:** Minor changes to the keys.

Groups	Key		
	$x_{0}^{\prime }$	$y_{0}^{\prime }$	$z_{0}^{\prime }$
Key_1_	0.01 + 10^−12^	0.01	0.01
Key_2_	0.01	0.01 + 10^−12^	0.01
Key_3_	0.01	0.01	0.01 + 10^−12^

**Table 8:** NPCRs and UACIs for different key groups.

Key Group	Key_1_	Key_2_	Key_3_
NPCR(%)	99.5956	99.6124	99.5697
UACI(%)	33.3818	33.5267	33.3440

**Table 9:** Correlations of adjacent pixels in grayscale images.

Images	Plain Image			Cipher Image		
	Horizontal	Vertical	Diagonal	Horizontal	Vertical	Diagonal
Baboon	0.8244	0.8750	0.7873	−0.0030	0.0131	0.0072
Boat	0.9457	0.9277	0.8895	0.0096	−0.0026	0.0179
Cameraman	0.9550	0.9224	0.9009	0.0211	0.0044	−0.0005
House	0.9673	0.9781	0.9521	−0.0092	0.0016	−0.0066
Peppers	0.9686	0.9617	0.9360	−0.0002	−0.0084	−0.0012

**Table 10:** NPCR and UACI results of the differential attack.

Images	Baboon	Boat	Cameraman	House	Peppers
NPCR(%)	99.6521	99.6002	99.5859	99.6445	99.6109
UACI(%)	33.6032	33.5662	33.6737	33.6547	33.4502

**Table 11:** PSNR values comparing the original and decrypted images after introducing salt-and-pepper noise.

Noise	0.01	0.05	0.1
PSNR	28.9271	22.3642	19.1878

**Table 12:** PSNR values for the original and decrypted images following cropping attacks.

Cropping	1/64	1/16	1/4
PSNR	47.9517	33.8976	21.2934

**Table 13:** Results of the chi-square test.

Image	Baboon	Boat	Cameraman	House	Peppers
Plain image	79,056.91	100,853.5	161,271.88	299,789.23	31,629.66
Cipher image	245.2344	260.4766	222.7813	278.7969	230.9922

**Table 14:** Information entropy of various images.

Image	Baboon	Boat	Cameraman	House	Peppers
Plain image	7.0092	7.1572	6.9046	6.4971	7.5797
Cipher image	7.9973	7.9971	7.9975	7.9970	7.9975

**Table 15:** Local information entropy of different images.

Image	Baboon	Boat	Cameraman	House	Peppers
Plain image	6.7229	6.3100	5.9547	5.3629	6.9630
Cipher image	7.9030	7.9027	7.9033	7.9033	7.9030

**Table 16:** Statistical analysis of the encrypted all black and all white images.

Information Entropy	Chi-Square Test	Correlation Coefficients			Information Entropy	Local Information Entropy
		Horizontal	Vertical	Diagonal		
All black	249.3203	0.0061	−0.0044	0.0165	7.9973	7.9039
All white	261.4062	0.0063	−0.0104	−0.0028	7.9972	7.9000

**Table 17:** Analysis of differential attacks on the encrypted all-black and all-white images.

Images	All Black	All White
NPCR (%)	99.6597	99.6201
UACI (%)	33.5505	33.5123

**Table 18:** Comparison with other studies.

	Correlation Coefficients			Information Entropy	NPCR (%)	UACI (%)
	Horizontal	Vertical	Diagonal			
Proposed	−0.0002	0.0084	−0.0012	7.9975	99.6109	33.4501
Ref. [38]	0.0010	−0.0040	0.0079	7.9971	99.6154	33.5456
Ref. [39]	0.0151	−0.0038	−0.0029	7.9969	99.6038	33.4974
Ref. [40]	−0.0016	0.0018	−0.0078	7.9972	99.5987	33.5056

**Table 19:** Comparison of encryption and decryption times for different algorithms.

	Size	Encryption Time (s)
Proposed	256 × 256	0.8253
Ref. [38]	256 × 256	0.7091
Ref. [41]	256 × 256	4.688
Ref. [42]	256 × 256	1.61

## Data Availability

The original contributions presented in the study are included in the article, further inquiries can be directed to the corresponding authors.
